# Supplementary material for: Hawks steer attacks using a guidance system tuned for close pursuit of erratically manoeuvring targets
Source: Nat Commun. 2019 Jun 11;10:2462. doi: 10.1038/s41467-019-10454-z (PMC6560099; doi:10.1038/s41467-019-10454-z)
Supplement: Supplementary file 2 — Supplementary Information [file 41467_2019_10454_MOESM2_ESM.pdf]

Supplementary Information for

Hawks steer attacks using a guidance system tuned for close pursuit of erratically maneuvering targets

Caroline H. Brighton, Graham K. Taylor

Correspondence to: graham.taylor@zoo.ox.ac.uk and caroline.brighton@zoo.ox.ac.uk

This PDF file includes:

Supplementary Figures 1 to 9
Supplementary Tables 1 and 2

Other Supplementary Materials for this manuscript include the following:

Supplementary Movies 1 and 2
Supplementary Data 1

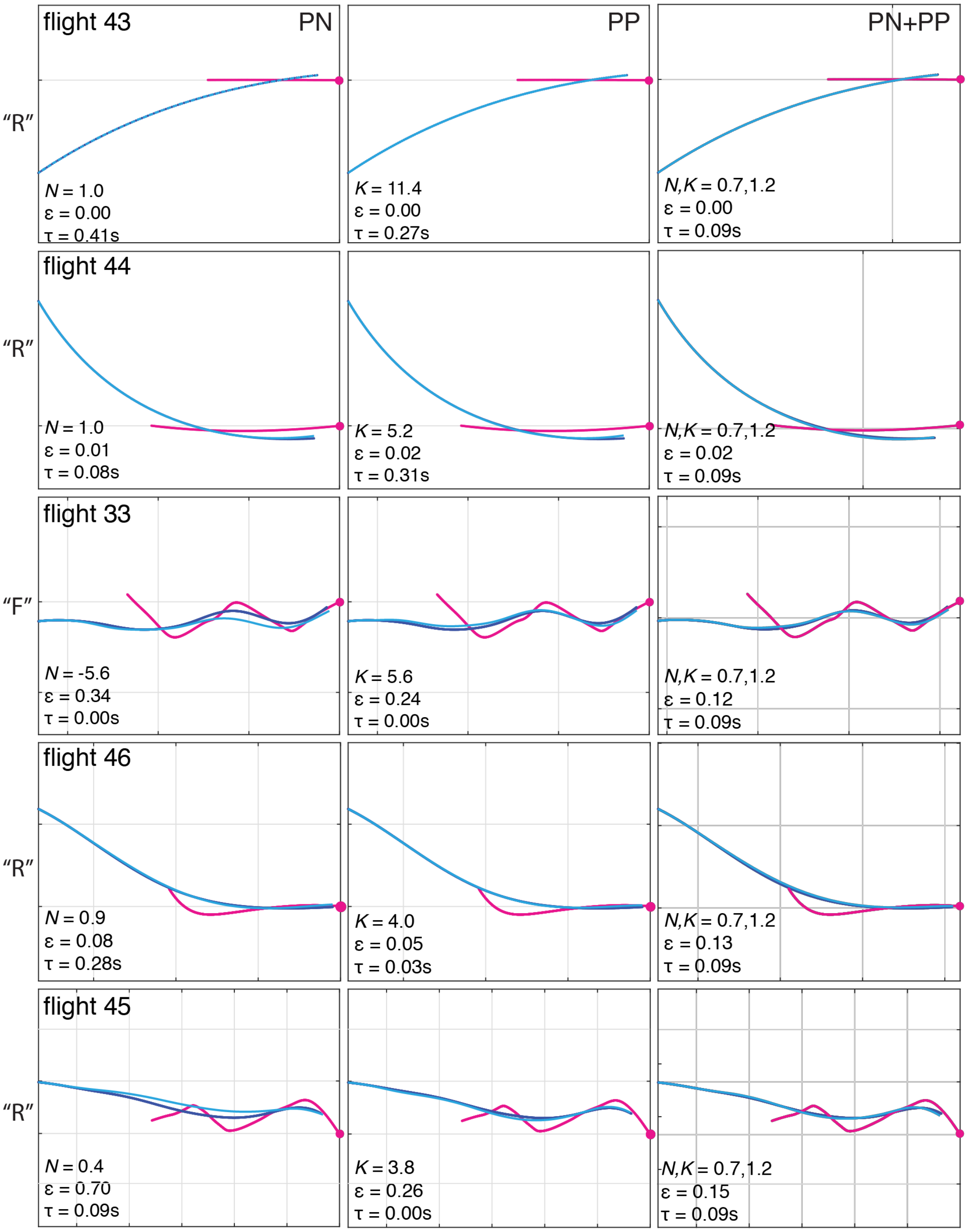

Supplementary Figure 1. Comparison of measured and simulated attack trajectories. Panels display measured attack trajectories (dark blue) and best-fitting simulations (light blue) for each Harris' Hawk in pursuit of the lure (magenta), arranged by individual bird (rows) and by guidance law (columns; PN: proportional navigation; PP: proportional pursuit; PN+PP: mixed guidance law). The time delay $\boldsymbol{\tau}$ (s) and guidance constant $\boldsymbol{K}$ (s^{-1}) or $\boldsymbol{N}$ are independently-fitted to each flight for PP and PN, but are globally-fitted to all flights for the mixed guidance law. Grid spacing: 10m. For flights fitted more closely by mixed guidance law, see Fig. 4; for flights fitted less closely, see Supplementary Figs. 2-9.

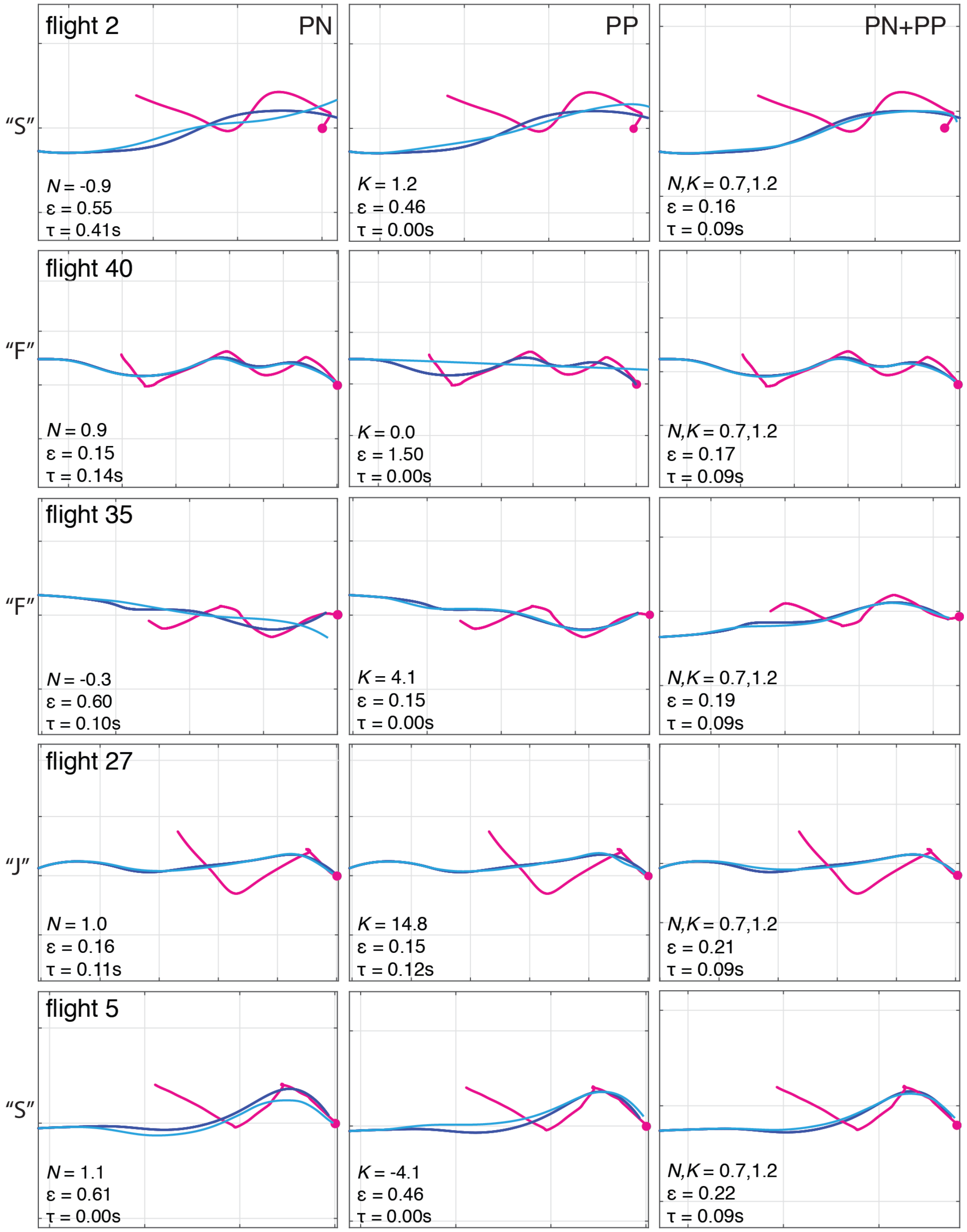

Supplementary Figure 2. Comparison of measured and simulated attack trajectories. Panels display measured attack trajectories (dark blue) and best-fitting simulations (light blue) for each Harris' Hawk in pursuit of the lure (magenta), arranged by individual bird (rows) and by guidance law (columns; PN: proportional navigation; PP: proportional pursuit; PN+PP: mixed guidance law). The time delay $\boldsymbol{\tau}$ (s) and guidance constant $\boldsymbol{K}$ (s^{-1}) or $\boldsymbol{N}$ are independently-fitted to each flight for PP and PN, but are globally-fitted to all flights for the mixed guidance law. Grid spacing: 10m. For flights fitted more closely by mixed guidance law, see Fig. 4 and Supplementary Fig. 1; for those fitted less closely, see Supplementary Figs. 3-9.

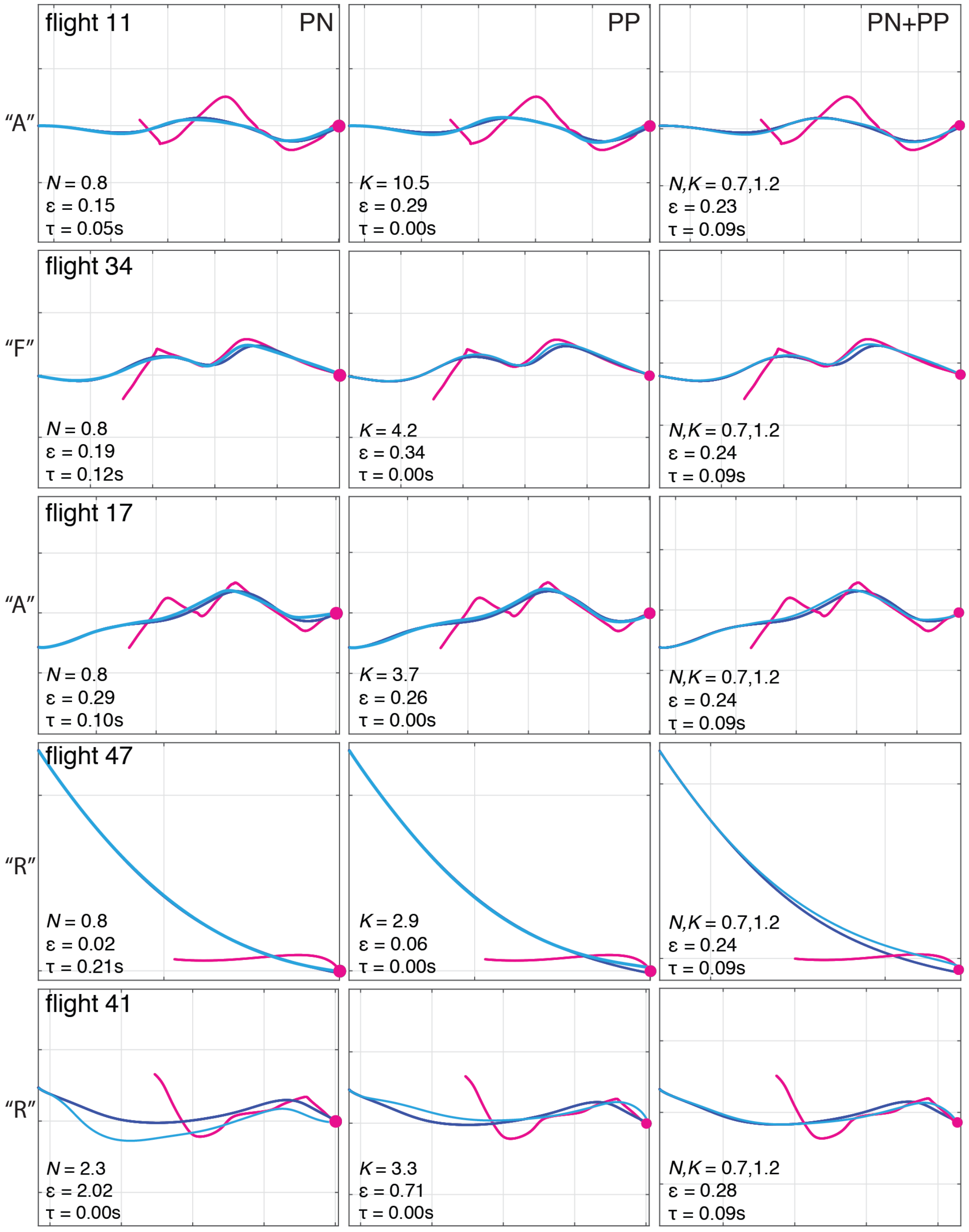

Supplementary Figure 3. Comparison of measured and simulated attack trajectories. Panels display measured attack trajectories (dark blue) and best-fitting simulations (light blue) for each Harris' Hawk in pursuit of the lure (magenta), arranged by individual bird (rows) and by guidance law (columns; PN: proportional navigation; PP: proportional pursuit; PN+PP: mixed guidance law). The time delay τ (s) and guidance constant K (s^{-1}) or N are independently-fitted to each flight for PP and PN, but are globally-fitted to all flights for the mixed guidance law. Grid spacing: 10m. For flights fitted more closely by mixed guidance law, see Fig. 4 and Supplementary Figs. 1-2; for those fitted less closely, see Supplementary Figs. 4-9.

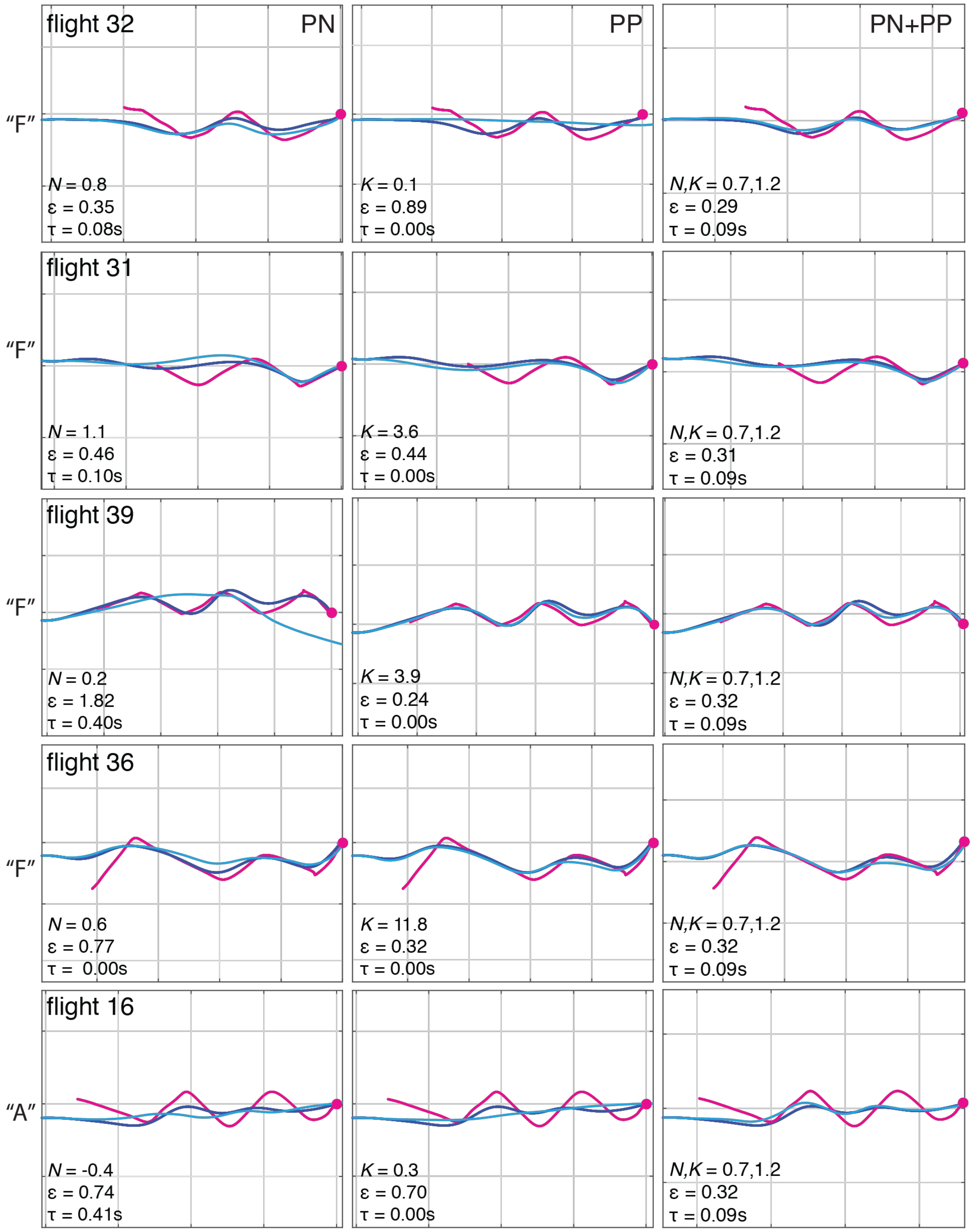

Supplementary Figure 4. Comparison of measured and simulated attack trajectories. Panels display measured attack trajectories (dark blue) and best-fitting simulations (light blue) for each Harris' Hawk in pursuit of the lure (magenta), arranged by individual bird (rows) and by guidance law (columns; PN: proportional navigation; PP: proportional pursuit; PN+PP: mixed guidance law). The time delay τ (s) and guidance constant K (s^{-1}) or N are independently-fitted to each flight for PP and PN, but are globally-fitted to all flights for the mixed guidance law. Grid spacing: 10m. For flights fitted more closely by mixed guidance law, see Fig. 4 and Supplementary Figs. 1-3; for those fitted less closely, see Supplementary Figs. 5-9.

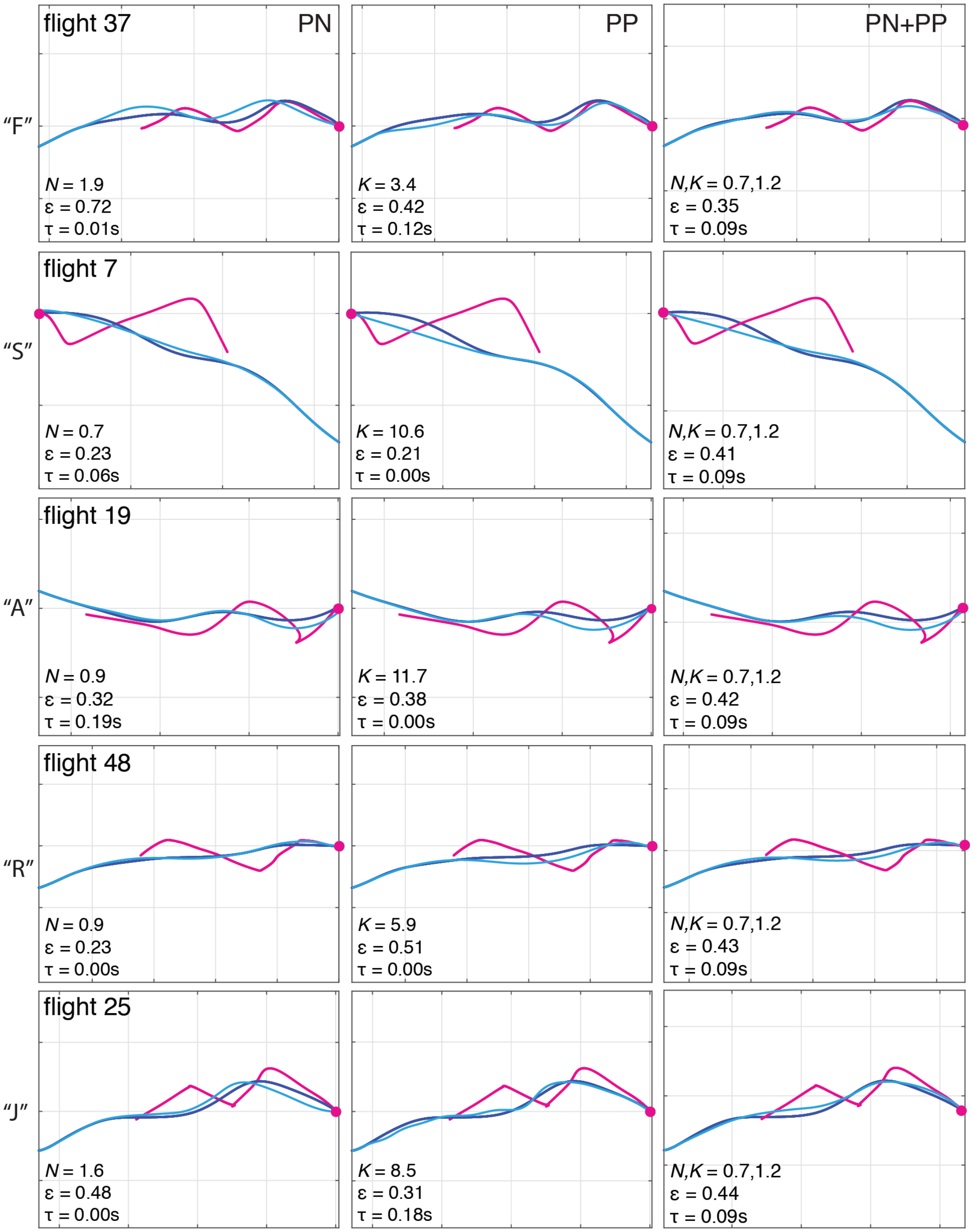

Supplementary Figure 5. Comparison of measured and simulated attack trajectories. Panels display measured attack trajectories (dark blue) and best-fitting simulations (light blue) for each Harris' Hawk in pursuit of the lure (magenta), arranged by individual bird (rows) and by guidance law (columns; PN: proportional navigation; PP: proportional pursuit; PN+PP: mixed guidance law). The time delay τ (s) and guidance constant K (s^{-1}) or N are independently-fitted to each flight for PP and PN, but are globally-fitted to all flights for the mixed guidance law. Grid spacing: 10m. For flights fitted more closely by mixed guidance law, see Fig. 4 and Supplementary Figs. 1-4; for those fitted less closely, see Supplementary Figs. 6-9.

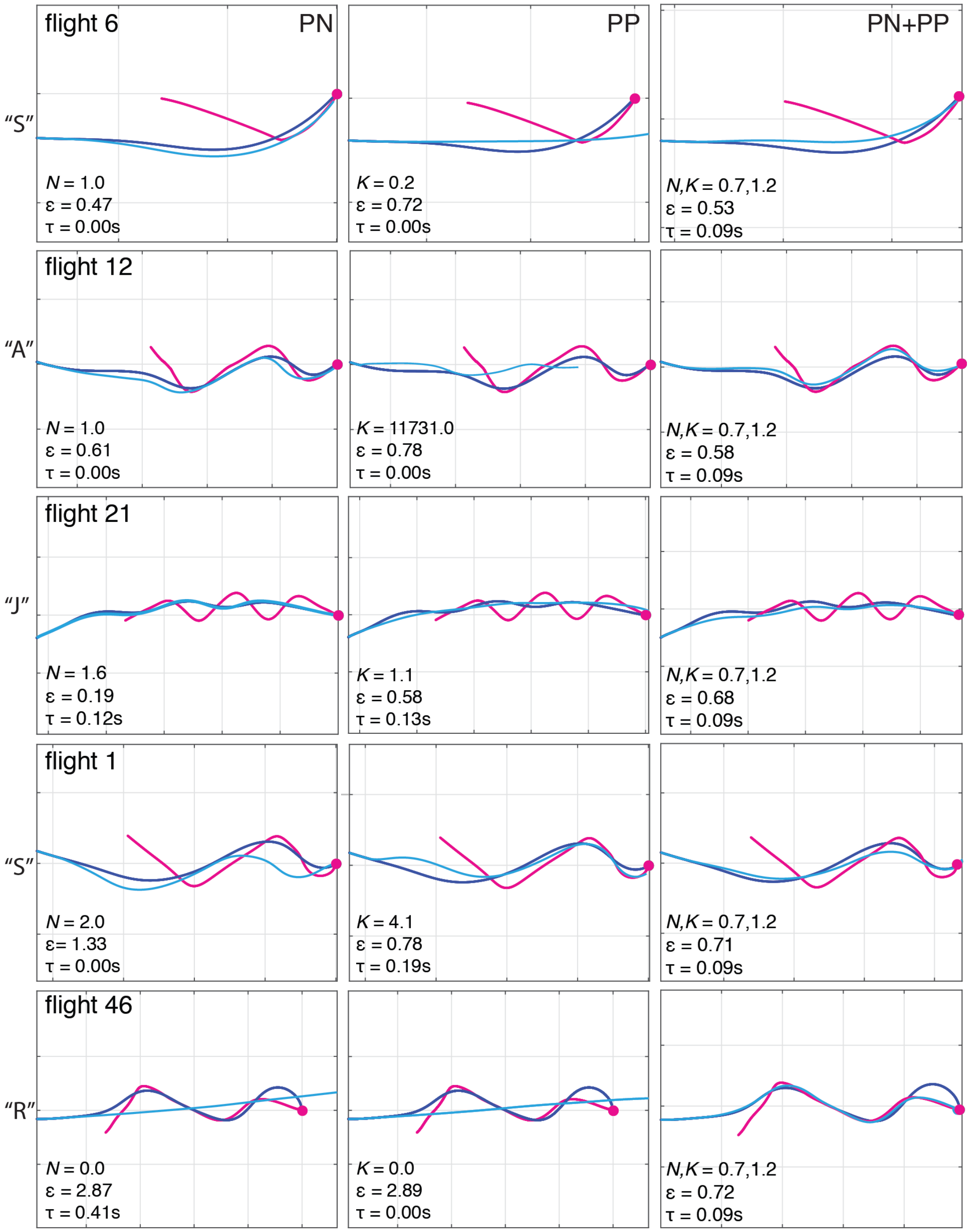

Supplementary Figure 6. Comparison of measured and simulated attack trajectories. Panels display measured attack trajectories (dark blue) and best-fitting simulations (light blue) for each Harris' Hawk in pursuit of the lure (magenta), arranged by individual bird (rows) and by guidance law (columns; PN: proportional navigation; PP: proportional pursuit; PN+PP: mixed guidance law). The time delay τ (s) and guidance constant K (s^{-1}) or N are independently-fitted to each flight for PP and PN, but are globally-fitted to all flights for the mixed guidance law. Grid spacing: 10m. For flights fitted more closely by mixed guidance law, see Fig. 4 and Supplementary Figs. 1-5; for those fitted less closely, see Supplementary Figs. 7-9.

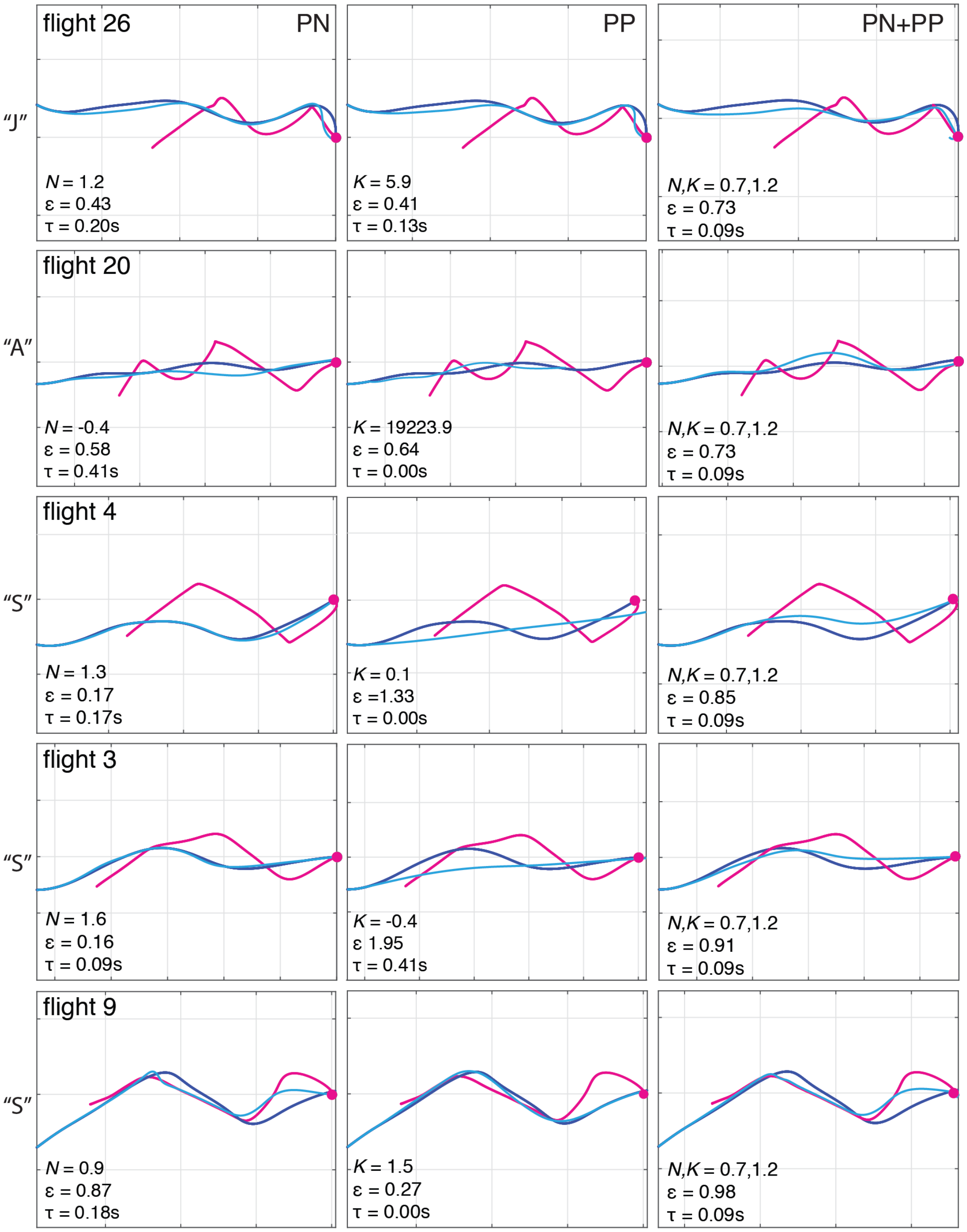

Supplementary Figure 7. Comparison of measured and simulated attack trajectories. Panels display measured attack trajectories (dark blue) and best-fitting simulations (light blue) for each Harris' Hawk in pursuit of the lure (magenta), arranged by individual bird (rows) and by guidance law (columns; PN: proportional navigation; PP: proportional pursuit; PN+PP: mixed guidance law). The time delay τ (s) and guidance constant K (s^{-1}) or N are independently-fitted to each flight for PP and PN, but are globally-fitted to all flights for the mixed guidance law. Grid spacing: 10m. For flights fitted more closely by mixed guidance law, see Fig. 4 and Supplementary Figs. 1-6; for those fitted less closely, see Supplementary Figs. 8-9.

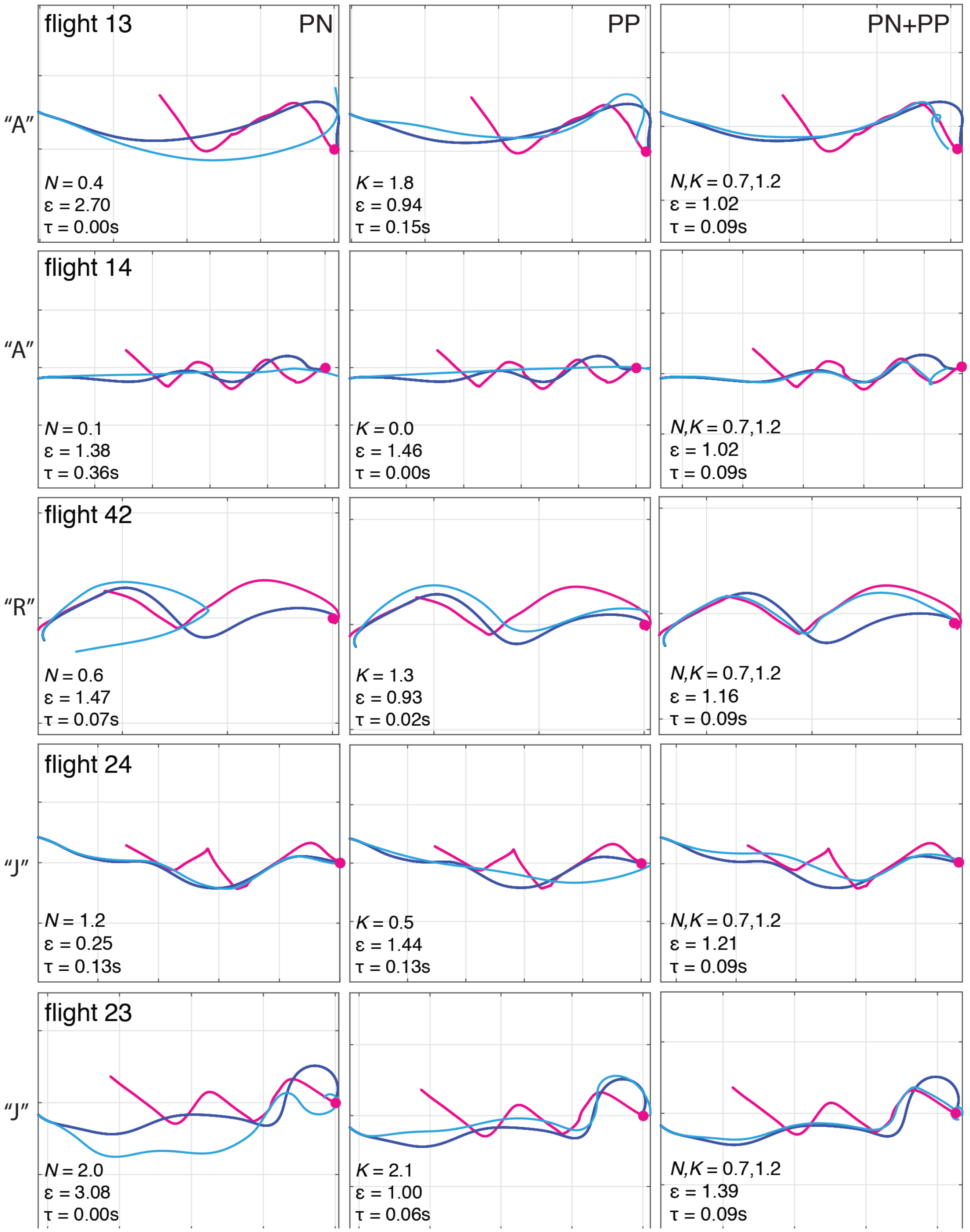

Supplementary Figure 8. Comparison of measured and simulated attack trajectories. Panels display measured attack trajectories (dark blue) and best-fitting simulations (light blue) for each Harris' Hawk in pursuit of the lure (magenta), arranged by individual bird (rows) and by guidance law (columns; PN: proportional navigation; PP: proportional pursuit; PN+PP: mixed guidance law). The time delay τ (s) and guidance constant K (s^{-1}) or N are independently-fitted to each flight for PP and PN, but are globally-fitted to all flights for the mixed guidance law. Grid spacing: 10m. For flights fitted more closely by mixed guidance law, see Fig. 4 and Supplementary Figs. 1-7; for those fitted less closely, see Supplementary Fig. 9.

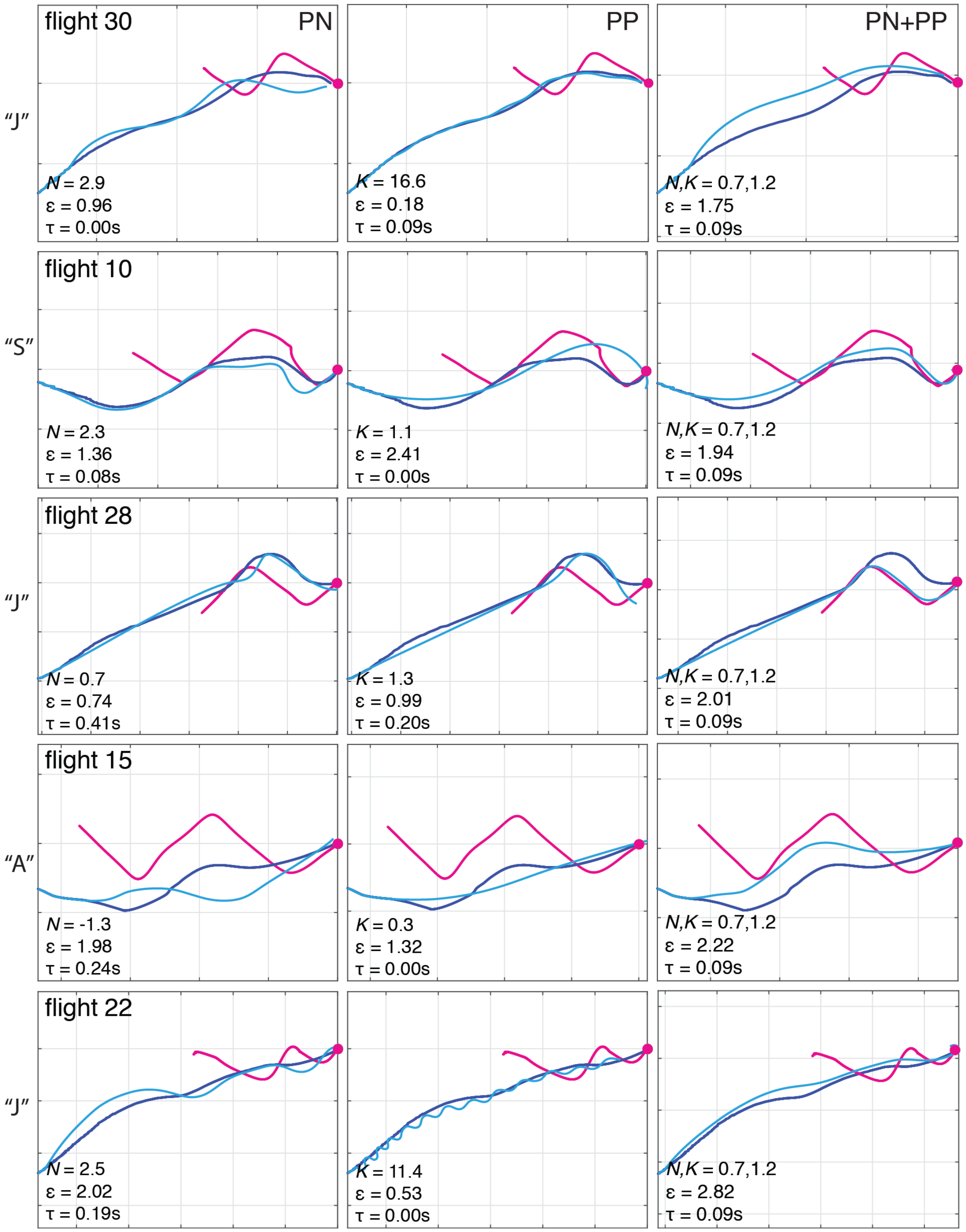

Supplementary Figure 9. Comparison of measured and simulated attack trajectories. Panels display measured attack trajectories (dark blue) and best-fitting simulations (light blue) for each Harris' Hawk in pursuit of the lure (magenta), arranged by individual bird (rows) and by guidance law (columns; PN: proportional navigation; PP: proportional pursuit; PN+PP: mixed guidance law). The time delay τ (s) and guidance constant K (s^{-1}) or N are independently-fitted to each flight for PP and PN, but are globally-fitted to all flights for the mixed guidance law. Grid spacing: 10m. These are the five flights that are least-well fitted by the mixed guidance law: for other flights, see Fig. 4 and Supplementary Figs. 1-8.

individual	code	sex	mass (kg)
“Spitfire”	“S”	male	0.66
“Aggy”	“A”	female	0.92
“Jake”	“J”	male	0.62
“Findo”	“F”	male	0.70
“Ruby”	“R”	female	0.93

Supplementary Table 1. Morphological data. Table containing information on the weight and sex of the $n = 5$ Harris’ Hawks *Parabuteo unicinctus* used in this study.

flight	bird	distance flown (m)	duration (s)	independently-fitted PP			independently-fitted PN			independently-fitted PP+PN				global PP+PN
				τ (s)	K (s^{-1})	ε (m)	τ (s)	N	ε (m)	τ (s)	K (s^{-1})	N	ε (m)	ε (m)
1	S	45.2	4.40	0.18	4.1	0.78	0.00	2.0	1.33	0.17	1.2	1.1	0.36	0.71
2	S	36.2	3.38	0.00	1.2	0.46	0.40	-0.9	0.55	0.12	1.4	0.9	0.09	0.16
3	S	55.0	5.45	0.40	-0.4	1.95	0.09	1.6	0.16	0.00	-0.6	1.6	0.18	0.91
4	S	41.5	4.20	0.00	0.1	1.33	0.16	1.3	0.17	0.13	0.2	1.3	0.04	0.85
5	S	32.4	3.24	0.00	4.1	0.46	0.00	1.1	0.61	0.16	1.0	0.8	0.02	0.22
6	S	28.9	2.92	0.00	0.2	0.72	0.00	1.0	0.47	0.12	0.6	1.1	0.02	0.53
7	S	36.2	2.99	0.00	10.6	0.47	0.06	0.7	0.23	0.10	-0.8	0.8	0.20	0.41
8	S	28.8	2.47	0.00	10.6	0.21	0.15	0.8	0.08	0.12	0.4	0.8	0.04	0.10
9	S	45.3	5.30	0.00	1.5	0.27	0.18	0.9	0.87	0.00	1.3	0.0	0.27	0.98
10	S	58.0	5.36	0.00	1.1	2.41	0.07	2.3	1.36	0.18	0.5	1.2	1.17	1.94
11	A	54.3	5.91	0.00	10.5	0.29	0.04	0.8	0.15	0.00	1.3	0.6	0.12	0.23
12	A	49.0	6.97	0.00	>100	0.78	0.00	1.0	0.61	0.06	0.2	0.7	0.28	0.58
13	A	47.5	6.15	0.15	1.8	0.94	0.00	0.4	2.70	0.10	0.9	0.2	0.43	1.02
14	A	52.4	7.93	0.00	0.0	1.46	0.35	0.1	1.38	0.32	-2.2	-0.3	0.90	1.02
15	A	46.1	4.61	0.00	0.3	1.32	0.24	-1.3	1.98	0.10	0.5	0.8	0.81	2.22
16	A	41.6	4.83	0.00	0.3	0.70	0.40	-0.4	0.74	0.13	0.6	0.6	0.14	0.32
17	A	52.9	6.43	0.00	3.7	0.26	0.10	0.8	0.29	0.00	2.5	0.3	0.19	0.24
18	A	36.2	4.68	0.00	1.7	0.38	0.00	2.7	0.99	0.22	1.0	0.8	0.06	0.21
19	A	34.8	3.36	0.00	11.7	0.38	0.19	0.9	0.32	0.17	-0.9	0.8	0.19	0.42
20	A	46.1	4.51	0.00	>100	0.64	0.40	-0.4	0.58	0.00	1.1	0.6	0.57	0.73
21	J	53.4	5.10	0.00	1.1	0.58	0.12	1.6	0.19	0.15	0.1	1.4	0.17	0.68
22	J	67.4	7.06	0.13	11.4	0.53	0.19	2.5	2.02	0.01	-0.7	3.4	1.86	2.82
23	J	51.5	6.19	0.06	2.1	1.00	0.00	2.0	3.08	0.28	0.7	0.5	0.60	1.39
24	J	52.8	5.83	0.00	0.5	1.44	0.13	1.2	0.25	0.09	-0.1	1.3	0.23	1.21
25	J	46.4	5.12	0.18	8.5	0.31	0.00	1.6	0.48	0.02	>100	-13.0	0.27	0.44
26	J	42.3	4.72	0.13	5.9	0.41	0.20	1.2	0.43	0.12	6.4	-0.1	0.41	0.73
27	J	52.3	4.18	0.12	14.8	0.15	0.11	1.0	0.16	0.10	4.1	0.8	0.15	0.21
28	J	70.2	6.14	0.20	1.3	0.99	0.40	0.7	0.74	0.40	0.5	0.5	0.74	2.01
29	J	55.3	5.48	0.00	6.1	0.16	0.00	1.4	0.87	0.02	2.4	0.5	0.10	0.18
30	J	41.2	3.00	0.09	16.6	0.18	0.00	2.9	0.96	0.01	>100	-10.8	0.16	1.75
31	F	43.0	5.50	0.00	3.6	0.44	0.10	1.1	0.46	0.17	0.5	0.8	0.23	0.31
32	F	42.9	5.75	0.00	0.1	0.89	0.07	0.8	0.35	0.16	0.5	0.9	0.14	0.29
33	F	32.8	4.22	0.00	5.6	0.24	0.00	0.8	0.34	0.14	1.1	0.7	0.09	0.12
34	F	50.9	5.93	0.00	4.2	0.34	0.12	0.8	0.19	0.14	-0.1	0.8	0.18	0.24
35	F	39.9	4.68	0.00	4.1	0.15	0.09	-0.3	0.60	0.03	2.8	0.3	0.12	0.19
36	F	51.8	6.00	0.00	11.8	0.32	0.00	0.6	0.77	0.09	2.1	0.7	0.27	0.32
37	F	43.6	5.10	0.12	3.4	0.42	0.00	1.9	0.72	0.22	1.3	0.6	0.28	0.35
38	F	34.0	3.98	0.00	5.3	0.09	0.18	0.8	0.07	0.00	2.8	0.3	0.06	0.07
39	F	55.3	7.02	0.00	3.9	0.24	0.39	0.2	1.82	0.35	0.0	0.6	0.65	0.32
40	F	58.0	6.67	0.00	0.0	1.50	0.14	0.9	0.15	0.13	1.3	0.6	0.15	0.17
41	R	44.0	4.44	0.00	3.3	0.71	0.00	2.3	2.02	0.05	1.3	1.0	0.13	0.28
42	R	32.4	4.82	0.02	1.3	0.93	0.06	0.6	1.47	0.32	0.7	0.3	0.59	1.16
43	R	5.7	0.55	0.27	11.4	0.00	0.40	1.0	0.00	0.40	5.2	0.5	0.00	0.00
44	R	9.6	1.01	0.30	5.2	0.02	0.08	1.0	0.01	0.13	-13.8	3.5	0.00	0.02
45	R	54.7	4.52	0.00	3.8	0.26	0.09	0.4	0.70	0.04	1.4	0.5	0.11	0.15
46	R	38.8	3.52	0.02	4.0	0.05	0.27	0.9	0.08	0.02	4.1	0.0	0.05	0.13
47	R	21.9	2.09	0.00	2.9	0.06	0.20	0.8	0.02	0.00	1.3	0.4	0.02	0.24
48	R	49.3	4.28	0.00	5.9	0.51	0.00	0.9	0.23	0.00	0.3	0.8	0.19	0.43
49	R	55.9	5.62	0.00	0.0	2.89	0.40	0.0	2.87	0.38	-1.0	0.1	1.52	0.72
50	R	34.5	2.98	0.40	-0.4	1.01	0.15	0.8	0.05	0.07	1.2	0.6	0.05	0.06

Supplementary Table 2. Summary of model parameters and fit. Table detailing the best-fitting simulations fitted under the independently-fitted proportional pursuit (PP), proportional navigation (PN), and mixed (PP+PN) guidance laws minimizing the prediction error for each flight separately; τ: delay; K and N: guidance constants; ε: prediction error, defined as the mean absolute distance between the measured and simulated trajectories. The last column of the table details the prediction error under the globally-fitted mixed guidance law minimizing the median prediction error over all of the flights, with model parameters $\tau = 0.09$ s, $K = 1.2$ s^{-1}, and $N = 0.7$ for each flight.
