# Supplementary material for: Hawks steer attacks using a guidance system tuned for close pursuit of erratically manoeuvring targets
Source: Nat Commun. 2019 Jun 11;10:2462. doi: 10.1038/s41467-019-10454-z (PMC6560099; doi:10.1038/s41467-019-10454-z)
Supplement: Supplementary file 4 — Description of Additional Supplementary Files [file 41467_2019_10454_MOESM4_ESM.docx]

**Description of Additional Supplementary Files**

**File Name: Supplementary Movie 1**

**Description:** Sample high-speed video of an attack recorded at 250fps.

**File Name: Supplementary Movie 2**

**Description:** Sample high-speed video of an attack recorded at 250fps.

**File Name: Supplementary Data 1**

**Description:** Zipped folder containing raw trajectory data and code for analysis. See the

README file within folder for details of code.
